# *ABCB4* variant is associated with hepatobiliary MR abnormalities in people with low-phospholipid-associated cholelithiasis syndrome

**DOI:** 10.1016/j.jhepr.2022.100590

**Published:** 2022-09-21

**Authors:** Moustafa Biyoukar, Christophe Corpechot, Sanaâ El Mouhadi, Edouard Chambenois, Quentin Vanderbecq, Véronique Barbu, Catherine Dong, Sara Lemoinne, Mickael Tordjman, Raphel Jomaah, Olivier Chazouilleres, Lionel Arrivé

**Affiliations:** 1 Department of Radiology, Saint-Antoine Hospital, Assistance Publique – Hôpitaux de Paris (APHP) and Sorbonne University, Paris, France; 2 Reference Center for Inflammatory Biliary Diseases and Autoimmune Hepatitis, ERN Rare-Liver, Department of Hepatology, Saint-Antoine Hospital, Assistance Publique – Hôpitaux de Paris (APHP), and INSERM, UMRS 938 Sorbonne University, Paris, France; 3 Department of Radiology, Raymond Poincaré Hospital, Assistance Publique – Hôpitaux de Paris (APHP), Garches, France; 4 Faculty of Arts and Science, University of Toronto, Toronto, ON, Canada

**Keywords:** LPAC, *ABCB4*, MDR3, Cholelithiasis, MRCP, Ursodesoxycholic acid, 3D, 3-dimensional, *ABCB4*, ATP-binding-cassette subfamily B, member 4, GGT, gamma-glutamyl transferase, LPAC, low-phospholipid-associated cholelithiasis, MDR3, multidrug resistance protein 3, MR, magnetic resonance, MRC, MR cholangiography, MRI, magnetic resonance imaging, OR, odds ratio, PACS, picture archive and communication system, UDCA, ursodeoxycholic acid, US, ultrasound

## Abstract

**Background & Aims:**

The low-phospholipid-associated cholelithiasis (LPAC) syndrome is a recently described peculiar form of cholelithiasis associated with the ATP-binding-cassette subfamily B, member 4 (*ABCB4*) gene deficiency. The purpose of our study was to analyse the relationship between magnetic resonance (MR) features and the genetic status of *ABCB4* in people with LPAC syndrome.

**Methods:**

A total of 233 individuals with proven LPAC syndrome were enrolled between January 2003 and June 2018 in a retrospective single-centre study. Inclusion criteria included availability of clinical files, MR images, and genetic data. MR images were analysed by consensus among 3 senior radiologists blinded to the status of *ABCB4* gene mutation.

**Results:**

A total of 125 individuals (mean age at first MR imaging 40.8 years; 66% females; 48% *ABCB4* variant) were included. MR abnormalities were found in 61 (49%) of the 125 individuals. Forty (67%) of the 60 individuals with an *ABCB4* gene variant had MR abnormalities as compared with 21 (33%) of the 65 individuals without an *ABCB4* gene variant (odds ratio [OR] 4.1, 95% CI 1.9–9.5, *p* = 0.0001). Compared to individuals with no variant, individuals with an *ABCB4* variant were more likely to show intrahepatic macrolithiasis (56 vs. 17%; OR 6.3, 95% CI 2.6–16.2, *p* <0.0001), bile duct dilatation (60 vs. 18%; OR 6.5, 95% CI 2.7–16.3, *p* <0.0001), and at least 1 MR feature of complication (35 vs. 15%; OR 2.9, 95% CI 1.1–7.8, *p* <0.05).

**Conclusions:**

*ABCB4*-related LPAC syndrome is associated with more frequent and severe hepatobiliary MR abnormalities. This finding strongly supports the major role of the *ABCB4* gene in the pathogenesis of LPAC syndrome and highlights a genotype–phenotype association in this inherited disease with genetic heterogeneity.

**Lay summary:**

*ABCB4*-related LPAC syndrome associated with an *ABCB4* gene variant demonstrates more frequent and severe hepatobiliary MR abnormalities. This finding supports the major role of the *ABCB4* gene in the pathogenesis of LPAC syndrome.

## Highlights

• We analysed the relationship between MRI and the genetic status of *ABCB4* in LPAC.
• MR abnormalities were found in 61 (49%) of the 125 individuals with LPAC syndrome.
• Forty (67%) of the 60 individuals with an *ABCB4* gene variant had MR abnormalities.
• Twenty-one (33%) of the 65 individuals without an *ABCB4* gene variant had MR abnormalities.
• *ABCB4*-related LPAC is associated with more frequent and severe MR abnormalities.

## Graphical abstract

**Figure:**
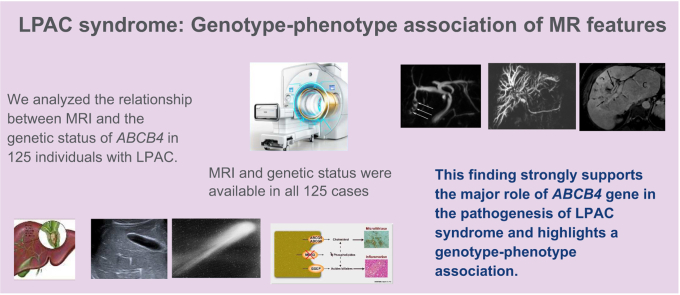

## Introduction

Low-phospholipid-associated cholelithiasis (LPAC) syndrome is a recently described rare form of cholelithiasis that has been linked to the ATP-binding-cassette subfamily B, member 4 (*ABCB4*) gene deficiency.^1^ This condition mostly affects young adults and should be suspected when at least 2 of the following clinical features are present: age at onset of biliary symptoms under 40 years; recurrence of biliary symptoms after cholecystectomy; and hyperechoic intrahepatic foci or comet tail images within intrahepatic bile ducts.^2^

*ABCB4* gene encodes the multidrug resistance protein 3 (MDR3).^1^ This protein, whose expression is potentially altered in LPAC syndrome, is a membrane transporter involved in the secretion of phospholipids and solubilisation of cholesterol into bile and the protection of biliary epithelium from bile acids toxicity. Deficit in MDR3 results in an altered bile composition, cholesterol crystal formation, and bile duct luminal membrane injuries by defective neutralisation of hydrophobic endogenous bile acids.

The first report by Rosmorduc *et al.*^1^ suggested a link between this genetic disorder and LPAC syndrome. Since then, several studies reported a percentage of approximately 30–50% of individuals with LPAC syndrome exhibiting *ABCB4* mutation, thus indicating a genetic heterogeneity of the disease.[3], [4], [5], [6] Our group recently found *ABCB4* gene variations in 122 (45%) out of 269 individuals with LPAC syndrome, most of those being single allele, missense variants.^7^ However, genetic polymorphism is of prime importance, and various types of variations have been reported, including nonsense mutation, missense mutation, and partial gene deletion.^4^ LPAC syndrome is also characterised by radiological polymorphism. Whereas the majority of individuals present with a typical microlithiasis form of the disease limited to multiple comet tail images on ultrasound (US) but normal cholangiogram on MR imaging (MRI), a significant proportion of them has abnormal MR features with intrahepatic macrolithiasis and focal biliary dilatation.^7^

The purpose of our study was to describe the MR features and to analyse their relationship with the genetic status of *ABCB4* in individuals with LPAC syndrome.

## Materials and methods

Our local institution review board approved the review of radiological and clinical data for this study. Informed consent was obtained from all participants.

### Study population

We retrospectively reviewed the database of prospectively included individuals with proven LPAC syndrome who were referred to our tertiary care centre between January 2003 and June 2018. We enrolled individuals with confirmed diagnosis of LPAC syndrome, known *ABCB4* gene status, and available MRI with at least 3-dimensional (3D) MR cholangiography (MRC) and T2-weighted images. Time of inclusion was defined by the first available MRI.

The diagnosis of LPAC syndrome was based on the presence of at least 2 of the 3 pre-established criteria:^7^ (i) onset of biliary symptoms before the fourth decade; (ii) recurrence of biliary symptoms after cholecystectomy; (iii) intrahepatic microlithiasis characterised by comet-tail images or hyperechoic foci on an ultrasonography performed by an experienced radiologist.

### Imaging techniques and analysis

All included individuals underwent MRI with at least 3D MRC and T2-weighted images.^8^ Additional sequences included the following: (i) fat-suppressed spin-echo T2-weighted sequence; (ii) in and opposed-phase gradient-echo T1-weighted axial plane sequence; (iii) transverse T1-weighted gradient-echo images with fat suppression before and after gadolinium chelates injection obtained at hepatic arterial, portal, and equilibrium phase acquisition (30 s, 90 s, and 3 min, respectively); and (iv) transverse diffusion weighted images with a b value of >400 s/mm^2^. Images were analysed by consensus among 3 senior radiologists; all readers were experienced in abdominal imaging with 30, 15, and 10 years of expertise. Assessment of imaging was blinded to the status of *ABCB4* gene. MRI from external centres were transmitted using compact discs and archived on a picture archive and communication system (PACS) workstation (version 11.32; Carestream Health, Rochester, NY, USA). MRIs from each participant taken during the follow-up period were analysed in chronological order using the PACS. Native images and 3D maximum intensity projection reconstructions were analysed on thick slabs of 10 or 20 mm oriented in the acquisition plane.

The morphological data analysed included the following:

A. MRC features

**Fig. 1:**
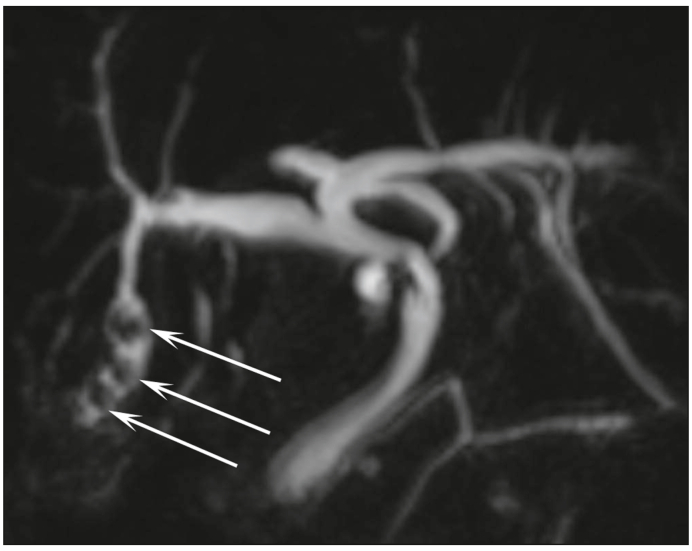
Biliary dilatation only observed at the level of stones (arrows).

**Fig. 2:**
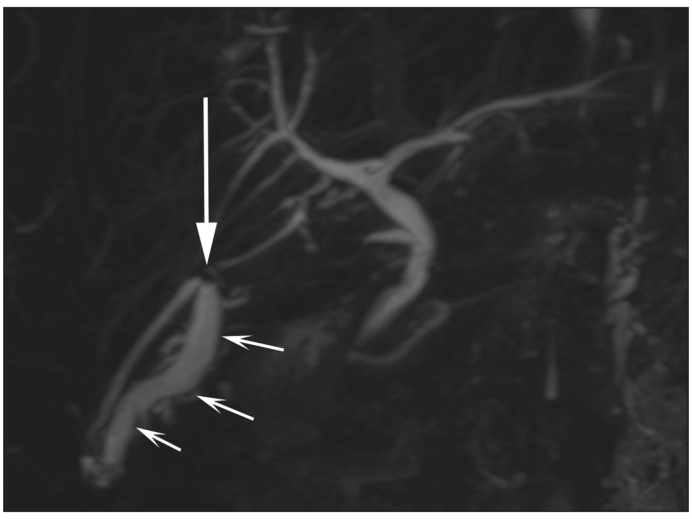
Biliary dilatation (small arrows) outside the level of stone (arrow).

These features pertain to the number, signal intensity, and location of stones in each segment of the liver. The signal intensity of stones was compared with the signal intensity of bile in T1- and T2-weighted images sequences (low signal intensity, high signal intensity, mixed [low and high signal intensity], and isosignal). Bile duct dilatations (intrahepatic bile duct >3 mm) are classified as follows (i) at the level of stones (*i.e.* biliary dilatation was only observed at the level of stones): (Fig. 1), (ii) outside the level of stones (Fig. 2), and (iii) with no visible stones.

B. MR features of complications

**Fig. 3:**
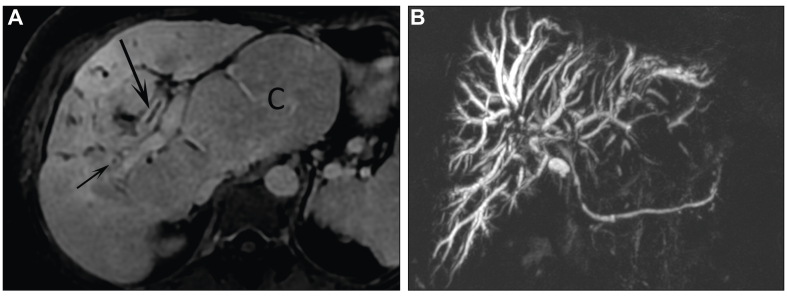
MR of biliary tree. (A) Contrast enhancement (arrows) of biliary walls and marked hepatic dysmorphia with hypertrophy of caudate (C) lobe. (B) Cholangitis-like multiple biliary stenoses.

These are complications such as bile duct abnormalities: contrast enhancement of biliary walls (Fig. 3A); stenosis (no stenosis/1 stenosis/several stenosis/cholangitis-like multiple stenosis; Fig. 3B); abscess formation suggested by small round peribiliary lesions with peripheral enhancement; associated liver-related signs including heterogeneous signal of hepatic parenchyma, loss of volume of the affected lobe or segment, dysmorphia (defined by analysis of modified caudate/right lobe ratio; Fig. 3A),^9^ and portal hypertension (defined by presence of portosystemic shunts); cholecystitis, pancreatitis; and cholangiocarcinoma.

As LPAC syndrome is defined by the presence of micro or macro intrahepatic stones, we considered that the impact on the bile ducts in the form of contrast enhancement or stenosis or on the hepatic parenchyma in the form of signal anomaly, loss of volume, or dysmorphia should be considered as complications. As a matter of fact, these different MR features have been considered as poor prognosis indicators in different prognostic scores developed with MRI for primary sclerosing cholangitis.^10^^,^^11^

C. MR follow-up

The inclusion criterion for the follow-up sub-study was the performance of at least 2 MRIs with at least 6 months apart between the 2 MRIs. Liver MR follow-up was evaluated and classified as follows:

• Improvement: reduction in the number of biliary stones or reduction in the dilation of the bile ducts without the appearance of any complication.
• Worsening: increase in the number of stones or worsening of dilatation of the bile ducts or appearance of a complication.
• Stability: neither improvement nor worsening.
D. US features

All US reports performed by an operator with extensive experience in LPAC US were reviewed for the presence of comet-tail images corresponding to twinkling artefacts resulting from US-induced vibration of cholesterol crystals and intrahepatic and extrahepatic stones.

### Genetic status

Genetic analysis was performed in all individuals to detect *ABCB4* gene mutation. Genomic DNA was obtained from peripheral white blood cells, and the identification of the *ABCB4* gene was achieved by Sanger sequencing (before 2014) or next-generation sequencing (from 2014 onwards).

The sequence variation was classified according to bioinformatics tables and literature data into the following categories:^4^^,^^7^^,^[12], [13], [14]

1. Disease-causing sequence variation (proven activity of the sequence variation on the MDR3 protein according to the bioinformatics tables and to literature).
2. Potentially pathogenic sequence variation (weak activity of the sequence variation on the MDR3 protein according to the bioinformatics tables and literature or sequence variation found in more than 5% of our patient files).
3. Sequence variation of unknown significance (sequence variation with no information in the literature).

### Statistical analysis

Descriptive statistical data were collected: mean and standard deviation (SD) for continuous variables, and number and percentage (%) for categorical variables. The groups were compared using the Χ^2^ test, or the Fisher exact test when appropriate, for categorical variables and the Mann–Whitney *U* test for continuous variables. A *p* value <0.05 was considered statistically significant. Statistical analyses were performed using SPSS (IBM SPSS Statistics for Windows, version 25.0; 2017, Armonk, MY, USA).

## Results

**Fig. 4:**
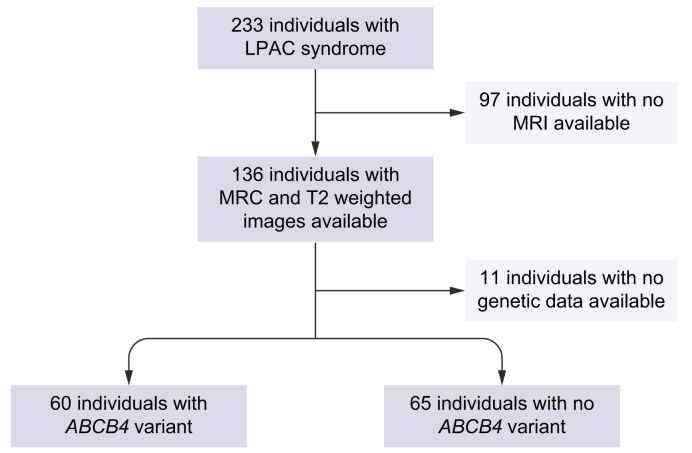
Flowchart of the study. *ABCB4*, ATP-binding-cassette subfamily B, member 4; LPAC, low-phospholipid-associated cholelithiasis; MRC, magnetic resonance cholangiography; MRI, magnetic resonance imaging.

After reviewing our series, 125 individuals were included (flowchart in Fig. 4).

Ninety-seven individuals in our cohort were excluded because MRI was not available. However, we did not find any significant difference in terms of age, sex ratio, history of cholecystectomy, treatment with ursodeoxycholic acid (UDCA), frequency, and characteristics of US abnormalities and genetic mutation between the groups with and without MRI.

Regarding inclusion criteria, 69 of the 125 individuals of our series fulfilled the 3 diagnosis criteria, and the other 56 individuals fulfilled 2 diagnosis criteria. Specifically, in our series of 125 individuals, ultrasonography was normal in 13 individuals, and there was no available expert US report in 13 other individuals.

In the group of 13 individuals with normal US, MRI was normal in 10 cases, and 7 of these 10 individuals were not mutated. In the group of 13 individuals without available expert US report, MRI was normal in 4 individuals, and 3 of these 4 individuals were not mutated.

Therefore, 10 individuals had no positive expert US result, had no MR abnormalities, and were not mutated. However, these 10 individuals presented with the 2 other major diagnosis criteria, and for 3 individuals, US result was not normal but unavailable.

Of the 125 individuals included, 60 (48%) had at least 1 *ABCB4* variant, and the other 65 (52%) had the wild-type gene (Fig. 4). In total, 312 MRC (171 MRC in individuals with a variant and 141 in individuals without) were reviewed (Table S1). The mean age at the onset of symptoms and at the first MRI were 31.1 ± 12.7 years (range 9–76 years) and 40.8 ± 15.8 years (range 14–81 years), respectively. A total of 121 (97%) of the 125 individuals who received UDCA started more than 1 year before the first MRI. Clinical and radiological features are presented in Table 1, Table 2, respectively.

**Table 1:** **Characteristics of individuals with LPAC according to *ABCB4* gene status**.

	*ABCB4* variant (n = 60)	No *ABCB4* variant (n = 65)	OR (95% CI)	*p* value
Sex, male/female	17/43	27/38	—	NS
Age at onset of symptoms	28.9	33.3	—	NS
Age at first MRI	38.8	43.8	—	NS
Cholecystectomy	47 (78%)	52 (80%)	—	NS
Chronic elevation of GGT (>2)	12 (20%)	14 (22%)	—	NS
MRI abnormalities	40 (67%)	21 (33%)	4.1 (1.9–9.5)	0.0001
Stones	34 (56%)	11 (17%)	6.3 (2.6–16.2)	<0.0001
Bile duct dilatation	36 (60%)	12 (18%)	6.5 (2.7–16.3)	<0.0001
Complications	21 (35%)	10 (15%)	2.9 (1.2–7.8)	<0.05

*ABCB4*, ATP-binding-cassette subfamily B, member 4; GGT, gamma-glutamyl transferase; LPAC, low-phospholipid-associated cholelithiasis; MRI, magnetic resonance imaging; OR, odds ratio.

**Table 2:** **MR abnormalities observed in 61 of 125 (49%) individuals**.

Bile duct stones	45/125 (36%)	
Signal of stones	**T1** = **42**	**T2** = **45**
Hyposignal	34 (81%)	44 (98%)
Hypersignal	3 (7%)	0 (0%)
Hyposignal and hypersignal	3 (7%)	1 (2%)
Isosignal	2 (6%)	0 (0%)
Number of stones		
1	4 (9%)	
2 or 3	5 (11%)	
Multiple	36 (80%)	
Location of stones		
Common bile duct	3 (7%)	
Intrahepatic bile duct	37 (82%)	
Diffuse	5 (11%)	
Number of liver segments affected		
1–2	28 (62%)	
3–4	12 (27%)	
≥5	5 (11%)	
Bile duct dilatation	48/125 (38%)	
At the level of stones	28 (58%)	
Outside the level of stones	15 (31%)	
With no stones	3 (6%)	
Cholangitis like	2 (4%)	
Complications	31 (25%)	
Contrast enhancement of biliary walls	3 (2%)	
Bile duct stenosis	3 (2%)	
Abscess	4 (3%)	
Heterogeneous signal of hepatic parenchyma	9 (7%)	
Loss of volume of hepatic segment	17 (14%)	
Dysmorphia	10 (8%)	
Portal hypertension	2 (2%)	
Cholecystitis	1 (1%)	
Pancreatitis	1 (1%)	
Cholangiocarcinoma	3 (2%)	

MRI, magnetic resonance.

### MR baseline data

MR abnormalities were found in 61 (49%) of the 125 individuals. Forty-five (36%) of these 125 individuals had 1 or more bile duct stones. Of those 45 individuals, 42 (93%) had intrahepatic stones, and 36 (80%) had multiple stones (*i.e.* >3). On T1-weighted images, intrahepatic stones presented low signal intensity in 81% (34 of 42 individuals with T1-weighted MRI) of cases. Forty-eight (38%) of these 125 individuals had at least 1 bile duct dilatation. Bile duct dilatation was only observed at the level of stones in 28 (58%) and outside the level of stones in 15 (31%) of those 48 individuals. MR features of complications were found in 31 (25%) of the 125 individuals (Table 2).

We observed 3 cases of cholangiocarcinoma in our series. All these 3 cases were mass-forming cholangiocarcinoma developed in symptomatic intrahepatic macrolithiasis cholangiopathy in individuals with an *ABCB4* variant.

### MR follow-up data

Fifty-four (43%) of the 125 individuals were eligible for this analysis (Tables S1 and S2). The average follow-up was 54 months. Among these 54 individuals, 7 had normal imaging that remained normal during follow-up. For the other 47 individuals, there was an improvement in 14/47 (30%), including a complete disappearance of imaging abnormalities in 2 and 1 having had surgery (liver resection); a worsening in 12/47 (25%); and a stable imaging in 21/47 (45%).

### US *vs.* MRI

Comparisons between US and MR features in the 112 of 125 individuals with LPAC with an expert US report available are presented in Table 3. In all cases, the time interval between US and MRI was less than 3 months. For the 112 individuals included, US abnormalities were found in 99/112 (88%), including comet tails in 79/99 (80%), intrahepatic lithiasis in 43/99 (43%), and common bile duct lithiasis in 27/99 (27%). Among the 60 individuals who had a normal MRI, the most common US abnormality was the comet-tail image found in 47 (92%) of the 51 individuals with US abnormalities. Conversely, among the 52 individuals who had an abnormal MRI, intrahepatic stones were observed in 32 (67%) of the 48 individuals with US abnormalities (Table 3).

**Table 3:** **Comparison between US and MR features in 112 individuals with US report**.

US features	All (N = 112)	Abnormal MRI (n = 52)	Normal MRI (n = 60)
Normal US	13/112(12%)	3/52 (6%)	10/60 (17%)
US abnormalities	99/112 (88%)	48/52 (92%)	51/60 (85%)
Comet-tail images	79/99 (80%)	32/48 (67%)	47/51 (92%)
Intrahepatic stones	43/99 (43%)	32/48 (67%)	11/51 (22%)
Common bile duct stones	27/99 (27%)	21/48 (44%)	6/51 (12%)

MR, magnetic resonance; MRI, MR imaging; US, ultrasound.

### *ABCB4* gene status

Sixty (48%) of the 125 individuals exhibited an *ABCB4* variant, whereas 65 (52%) presented with the wild-type sequence of the *ABCB4* gene (Table S3). Heterozygous mutation was observed in 57 (95%) of the 60 individuals and homozygous mutation in 3 (5%). Among the 60 individuals with mutation, the sequence variation was considered disease-causing in 38 (63%) cases, potentially pathogenic in 13 (22%), and of unknown significance in 9 (15%). Regarding MR features, we did not find any difference between these patterns of *ABCB4* sequence variation. Among the individuals excluded from analysis owing to the lack of available MRI (n = 97), 31 (39%) exhibited an *ABCB4* variant, whereas 48 (61%) presented with the wild-type sequence of the *ABCB4* gene (no genotype available for 18 individuals). Included and excluded individuals did not differ statistically in terms of *ABCB4* variant frequency (odds ratio [OR] 1.4, 95% CI 0.77–2.64).

#### MR features according to *ABCB4* gene status

Forty (67%) of the 60 individuals with an *ABCB4* variant had MRI abnormalities as compared with 21 (33%) of the 65 individuals with no variant (OR 4.1, 95% CI 1.9–9.5, *p* = 0.0001; Table 1). Compared with individuals without an *ABCB4* variant, individuals with *ABCB4* mutation were more likely to have intrahepatic stones (56 *vs*. 17%; OR 6.3, 95% CI 2.6–16.2, *p* <0.0001), bile duct dilatation (60 *vs*. 18%, OR 6.5, 95% CI 2.7–16.3, *p* <0.0001), and at least 1 MR feature of complication (35 *vs*. 15%; OR 2.9, 95% CI 1.1–7.8, *p* <0.05). However, we did not observe any significant differences between groups regarding the course of radiological features during follow-up.

## Discussion

Of the 125 individuals (60 with *ABCB4* variant and 65 without) with LPAC syndrome included in this single-centre study, we found that 61 (49%) presented MRI abnormalities. This higher-than-expected percentage may be explained by of our tertiary recruitment (referral bias) together with the retrospective design of the study, as not all individuals with LPAC presenting with a typical microlithiasis form of the disease are offered MRI in initial workup (selection bias). Therefore, it is not possible to accurately estimate the percentage of abnormal MR examinations in individuals with LPAC in the general population from our results. In addition, definite indications of MRI are uneasy to determine. In our daily clinical practice, the reference imaging examination is ultrasonography performed by an experienced operator. MRI is not performed systematically; it is generally offered in the event of significant key clinical, liver tests or marked US abnormalities. We demonstrated that MRI was more often abnormal when the US has shown stones and, on the contrary, MRI was more often normal when the US has only shown comet-tail images.

Our results highlighted that individuals with a potentially pathogenic variant of *ABCB4* gene have more MRI abnormalities (67 *vs*. 33%; OR 4.1, 95% CI 1.9–9.5) and MR features of complications (35 *vs*. 15%; OR 2.9, 95% CI 1.1–7.8) than individuals with the wild-type gene. These findings definitely set the *ABCB4* gene as a major player in the pathogenesis of the disease and should encourage physicians to perform hepatobiliary MRI as part of the routine workup for LPAC syndrome associated with an *ABCB4* gene defect. The main MR features of LPAC syndrome included multiple intrahepatic stones and bile duct dilatations. Stones mostly presented with low signal intensity on T1- and T2-weighted images in 80 and 98% of cases, respectively.

### MR characteristics

Two radiological patterns of LPAC syndrome expression have been described, including the microlithiasis and macrolithiasis forms.^2^^,^^4^ Individuals that presented with normal MRI (n = 64) had the microlithiasis pattern of LPAC syndrome. The US analysis of these individuals found abnormalities in 85% of cases, revealing comet-tail images in 92% of those cases. Comet-tail images correspond to twinkling artefacts resulting from US-induced vibration of cholesterol crystals, which are signs of microlithiasis and cannot so far be detected by MRI.

Intrahepatic lithiasis is more common in Asia and is less frequently encountered in Western countries.^15^ Stones are classified as cholesterol or pigment stones, as described by the intrahepatic stone classification of the Japanese Study Group.^16^ Intrahepatic calculi are commonly brown pigment stones at pathological examinations.^17^ In individuals with LPAC, stones are usually referred to as cholesterol yellow stones.^18^ The characteristics of intrahepatic calculi on MRI rely on their chemical composition.^19^ Some investigators have reported spontaneous hyperintensity on T1-weighted images in hepatolithiasis.^20^^,^^21^ Although a few intrahepatic stones presented high signal intensity or isosignal intensity on T1-weighted images, in our study, stones commonly appeared as intraluminal defects on low signal intensity on both T1- and T2-weighted images. These findings are consistent with the fact that low signal intensity in intrahepatic duct stones on T1- and T2-weighted images corresponded to cholesterol stones. Moreover, 7% of intrahepatic calculi showed high signal intensity on T1-weighted images, probably brown mixed stones related to several factors such as bile infection and bilirubin deconjugation. Hypersignal intensity of pigment stones can be related to the paramagnetic effect of the degradation products, which, by shortening the relaxation time, produce increased signal strength on T1-weighted images unlike cholesterol calculi. Heterogeneous signal of the liver parenchyma, focal parenchyma atrophy, and eventually dysmorphia represent the long-term consequences of biliary duct obstruction on liver parenchyma resulting from the chronic history of intrahepatic biliary calculi.^18^^,^^22^ These abnormalities were very uncommon in our series of individuals with LPAC. We assume that the consequences on the hepatic parenchyma were limited because intrahepatic bile duct dilatation was commonly limited to the level of stones.

Finally, there was a 10-year diagnostic delay between the age of the first MRI scan and the age of onset of symptoms partly caused by a lack of knowledge of the pathology and its rarity. This delay could partly explain the relatively unusual rate (nearly half) of individuals with MRI abnormalities in this series. Recently, our group reported an approximately 1% prevalence rate of LPAC syndrome in adult individuals presenting with symptomatic gallstone disease.^7^

### Association of *ABCB4* gene status with MR features

More importantly, the present results reinforce the strong association of LPAC syndrome with an *ABCB4* gene defect through distinct imaging phenotypes. Individuals with an *ABCB4* variant presented with more MRI abnormalities and complications than individuals with no mutation. However, we did not find any difference regarding the pattern of *ABCB4* (nonsense *vs*. missense) variants. Nonetheless, the present study strongly supports the major role of *ABCB4* in the pathogenesis of LPAC syndrome. Very few studies compared individuals with LPAC with and those without *ABCB4* variation. Poupon *et al.*^4^ found no statistical difference between individuals with and those without *ABCB4* mutations with regard to common bile duct stones, gallbladder stones, bile duct dilatation, or biliary complications. However, this study was essentially based on retrospective analysis of US reports, whereas our analysis was based on a specific review of MR images.^4^ Our group recently reported a significantly higher risk of common bile duct lithiasis, chronic elevation of gamma-glutamyl transferase (GGT), and personal or family history of hepatobiliary cancer in individuals with *ABCB4* gene variation.^8^ Population-based, genetic studies in Iceland have shown a significant association between the presence of certain polymorphic variants of the *ABCB4* gene and the occurrence of primary cancers of the liver regardless of the existence of cirrhosis.^23^ Cholangiocarcinoma is the primary liver cancer most frequently associated with intrahepatic lithiasis.^24^ Three cases of cholangiocarcinoma were observed during the follow-up in our study, all cases being occurred in individuals with macrolithiasis cholangiopathy and *ABCB4* mutation. Tougeron *et al.*^25^ reported 2 cases of cholangiocarcinoma in 2 independent adult individuals associated with MDR3 deficiency. Vij *et al.*^26^ also suggested a pathogenic role related to the MDR3 deficiency in a case of paediatric hepatocellular carcinoma. To date, however, no direct relationship has been established between genomic mutations and cholangiocarcinoma.^27^ It remains to be determined if tumorigenesis can occur independently of any parenchymal injury, in particular biliary cirrhosis.

Our study had limitations mainly related to its retrospective design from a single tertiary care centre. All individuals included underwent MRI and, therefore, as discussed above, may constitute a specific subgroup of LPAC syndrome. However, we did not find any significant difference in terms of *ABCB4* gene variation, clinical, biochemical, and US characteristics between included and excluded individuals because of lack of MRI. The second limitation was the referral bias in our tertiary care centre and, consequently, the process of reviewing from different institutions with heterogeneity of MRI protocols. Because of the retrospective nature of the study, contrast-enhanced sequences were not available for all individuals. In addition, in our series of 125 individuals, 10 had no positive expert US result, had no MR abnormalities, and were not mutated. Therefore, there is indeed a risk of overdiagnosis in these 10 individuals, which only represent 8% of our series. Finally, the genotype–phenotype relationship has been limited to *ABCB4*, and we did not investigate other biliary transporter genes, for example, *ABCB11*, *ATP8B1*, and *ABCG5*/*G8*. However, the pathogenicity of such other biliary transporter genes in LPAC syndrome remains to be established.

In conclusion, LPAC syndrome with an *ABCB4* variant is associated with more frequent and severe hepatobiliary MR abnormalities, highlighting for the first time a genotype–phenotype association in this disease. The main MR features in LPAC syndrome include the association of multiple low-signal-intensity intrahepatic stones with intrahepatic bile duct dilatations commonly limited to the level of stones with little consequences on the hepatic parenchyma.

## Financial support

No financial support was received for this study.

## Authors’ contributions

Have made substantial contributions to conception and design: MB, CC, CD, OC, LA. Were involved in design, acquisition of data, or analysis and interpretation of data: All authors. Were involved in drafting the manuscript or revising it: All authors. Approved the final version of the manuscript: All authors. Are accountable for accuracy and integrity of the article: All authors.

## Data availability statement

The data that support the findings of this study are available from the corresponding author (LA) upon reasonable request.

## Conflicts of interest

The authors declare that there is no conflict of interest.

Please refer to the accompanying ICMJE disclosure forms for further details.
