# Supplementary material for: ABCB4 variant is associated with hepatobiliary MR abnormalities in people with low-phospholipid-associated cholelithiasis syndrome
Source: JHEP Rep. 2022 Sep 21;4(11):100590. doi: 10.1016/j.jhepr.2022.100590 (PMC9582794; doi:10.1016/j.jhepr.2022.100590)
Supplement: Multimedia component 1 [file mmc1.pdf]

ABCB4 variant is associated with hepatobiliary MR abnormalities in people with low-phospholipid-associated cholelithiasis syndrome

Moustafa Biyoukar, Christophe Corpechot, Sanaâ El Mouhadi, Edouard Chambenois, Quentin Vanderbecq, Véronique Barbu, Catherine Dong, Sara Lemoinne, Mickael Tordjman, Raphel Jomaah, Olivier Chazouilleres, Lionel Arrivé

Table of contents

Table S1...2

Table S2...3

Table S3...4

Table S1: Number of MRCP in LPAC patients

Number of MRCP	No Variant (n=65)	Variant (n=60)	Total
1	42 (42)	27 (27)	69 (69)
2	8 (16)	10 (20)	18 (36)
3	3 (9)	5 (15)	8 (24)
4	4 (16)	8 (32)	12 (48)
5	3 (15)	0 (0)	3 (15)
More than 5	5 (43)	10 (77)	15 (120)

Table S2: Radiological follow up according to ABCB4 gene status

	ABCB4 variant (n= 31)	No ABCB4 variant (n= 16)	P
Improvement (14)	11 (35 %)	3 (18 %)	
Stability (21)	14 (45 %)	7 (43 %)	NS
Worsening (12)	6 (19 %)	6 (36 %)	

Table S3 - Genetic status in the 60 patients with ABCB4 variant

CASE	SEX	AGE	LOCATION AND NUCLEOTIDE CHANGES	SEQUENCE VARATIONS	IMPORTANCE OF SEQUENCE VARIATION *	STATUS
1	F	43	c.2800G>A	p.Ala934Thr	1	HTZ
2	F	24	c.140G>A & c.1217G>A	p.Arg47Gln & p.Arg406Gln	1	HTZ & HTZ
3	F	39	c.2800G>A	p.Ala934Thr	1	HTZ
4	M	51	c.857C>T	p.Ala286Val	2	HTZ
5	F	53	c.1769G>A	p.Arg590Gln	1	HTZ
6	F	64	c.2363G>A	p.Arg788Gln	2	HTZ
7	F	21	c.139C>T & c.823A>G	p.Arg47* & p.Thr175Ala	1	HTZ & HTZ
8	F	33	c.2869C>T	p.Arg957*	1	HTZ
9	F	34	c.3258C>A	p.Tyr1086*	1	HTZ
10	F	65	c.2471T>G	p.Val824Gly	2	HTZ
11	F	41	c;2363G>A	p.Arg788Gln	2	HTZ
12	M	76	c.1769G>A & c.2800G>A	p.Arg590Gln & p.Ala934Thr	1	HTZ & HTZ
13	F	28	c.1015dupT	p.Asp355*	1	HTZ
14	F	20	c.1015dupT	p.Asp355*	1	HTZ
15	F	23	c.344G>T	p.Arg115Ile	2	HTZ
16	F	38	c.1633C>T	p.Arg545Cys	1	HTZ
17	M	44	c.2661dup	p.Glu888Argfs*17	1	HTZ
18	M	34	c1646G>A	p.Arg549His	2	HTZ
19	M	44	c.475C>T	p.Arg159*	1	HTZ
20	F	31	c.1553delT	p.Leu518Tyrfs*16	1	HTZ
21	F	18	c.2363G>A	p.Arg788Gln	2	HMZ
22	M	39	c.536+6T>C	p.?	3	HTZ
23	F	44	c.140G>A	p.Arg47Gln	2	HTZ
24	M	23	c.523A>G & c.3269+1G>A	p.Thr175Ala & p.?	1	HTZ & HTZ
25	F	33	c.1712delT	p.Val571Aspfs*16	1	HTZ
26	F	38	c.959C>T	p.Ser320Phe	1	HTZ
27	M	47	c.498C>T & c.2078delC	p.Asp166Asp & p.Pro693Profs*5	1	HTZ
28	M	68	c.140G>A	p.Arg47Gln	2	HMZ
29	F	40	c.3838C>T	p.*1280Arg ext	1	HTZ
30	F	28	c.140G>A	p.Arg47Gln	1	HTZ
31	M	57	c.2324C>T & c.2836G>A	p.Thr775Met & p.Ala946Thr	1	HTZ & HTZ
32	M	29	c.2405G>A	p.Trp802*	1	HTZ
33	F	17	c.140G>A	p.Arg47Gln	2	HTZ
34	M	35	c.1576G>T	p.Val526Phe	1	HTZ
35	F	36	c.959C>T	p.Ser320Phe	1	HTZ
36	F	60	c.578G>A	p.Gly193Glu	2	HTZ
37	M	53	c.202G>A	p.Gly68Arg	1	HTZ

38	F	9	c.1296_1301del	p.Cys433_Gly434del	1	HTZ
39	M	29	c.1327insA	p.Cys433Argfs*7	1	HTZ
40	F	50	c.1896+6T>C	p.?	3	HMZ
41	F	35	c.79A>G	p.Ser27Gly	1	HTZ
42	F	51	c.2800G>A	p.Ala934Thr	1	HTZ
43	F	25	c.217C>G	p.Leu73Val	1	HTZ
44	M	14	c.523A>G	p.Thr175Ala	3	HTZ
45	F	51	c.1005+5G>A	p.?	3	HTZ
46	F	28	c.1778C>T	p.Thr593Met	1	HTZ
47	F	28	c.959C>T	p.Ser320Phe	1	HTZ
48	F	39	c.2800G>A	p.Ala934Thr	1	HTZ
49	F	41	c.3081+5G>C	p.?	3	HTZ
50	M	46	c.1584G>C	p.Glu528Asp	3	HTZ
51	F	48	c.1230+3A>C	p.?	3	HTZ
52	F	44	c.101C>T	p.Thr34Met	1	HTZ
53	F	36	c.1327C>G & c.1769G>A	p.Gln443Glu & p.Arg590Gln	1	HTZ & HTZ
54	F	35	c.2363G>A	p.Arg788Gln	2	HTZ
55	F	62	c.523A>G	p.Thr175Ala	3	HTZ
56	F	42	c.459T>C	p.Phe153Phe	3	HTZ
57	F	47	c.1769G>A	p.Arg590Gln	2	HTZ
58	M	38	c.524C>T	p.Thr175Met	1	HTZ
59	F	24	c.1675C>A	p.Ala559Thr	1	HTZ
60	F	38	c.1974G>A	p.Trp658*	1	HTZ

* 1: disease-causing ; 2: potentially pathogenic ; 3: of unknown significance
